# Preclinical to clinical translation of anti-PD-1 blockade

**DOI:** 10.1186/2051-1426-3-S2-P92

**Published:** 2015-11-04

**Authors:** Heather Hirsch, Elaine Pinheiro, Mark Ayers, Jared Lunceford, Michael Nebozhyn, Erin Murphy, Mingmei Cai, Yanhong Ma, Manjiri Sathe, Terri McClanahan

**Affiliations:** 1 Merck Research Laboratories, Boston, MA, USA; 2 Merck & Co., Inc., Kenilworth, NJ, USA; 3 Merck MRL, Lansdale, PA, USA; 4 Merck & Co., Inc., Palo Alto, CA, USA

## Background

Pembrolizumab (MK-3475), a humanized monoclonal IgG4 antibody against programmed death receptor 1 (PD-1), is currently being studied in clinical trials across more than 30 types of cancers. Immunotherapy with anti–PD-1 monoclonal antibodies such as pembrolizumab shows robust, durable anti-tumor activity in multiple indications including patients with advanced melanoma. A more complete understanding of the mechanism of action and biology associated with both response and resistance to pembrolizumab is critical to better inform on future clinical development and will also provide insight into the development of additional immuno-oncology therapies.

## Methods and results

To further support the clinical development of pembrolizumab, muDX400 (an anti-PD1 murine surrorgate) was tested in syngeneic mouse models. Response to muDX400 treatment in several syngeneic tumor models was broadly classified into 3 categories: highly responsive, partially responsive, and intrinsically resistant to therapy. Using a multifaceted approach, tumors from these models were extensively characterized at the molecular and cellular level by gene expression profiling and whole exome sequencing to help elucidate mechanisms of action and biology associated with response and resistance to anti-PD1 treatment.

These findings are also being compared to data that we are obtaining from ongoing clinical trials with pembrolizumab to help better understand the translatability of these preclinical models to the clinic. For example, both the IFN-γ and the expanded-immune signatures showed statistically significant associations with ORR and PFS in Keynote 001 Phase Ib melanoma trial. Analysis of top-ranked genes on the platform led to the discovery of two new signatures, “TCR-signaling” and “De novo” that were enriched in T cell markers and MHC Class I and II genes. Measuring immune-related biomarkers, including T cell specific, antigen presentation–related, and IFN-γ signaling–related genes, may allow for improved selection of patients likely to respond to anti–PD-1 therapy with pembrolizumab.

The translatability of molecular features such as gene expression and mutation burden will be assessed in syngeneic mouse models.

## Conclusions

These preclinical syngeneic tumor models allow us the opportunity to formulate and test specific hypotheses to more completely understand the biology behind the clinical successes currently observed with the novel cancer immunotherapies including anti-PD-1 antibodies such as pembrolizumab.

